# Non-closure of the Free Peritoneal Flap During Laparoscopic Hernia Repair of Lower Abdominal Marginal Hernia: A Retrospective Analysis

**DOI:** 10.3389/fsurg.2021.748515

**Published:** 2021-11-30

**Authors:** Qian Xu, Guangyong Zhang, Linchuan Li, Fengting Xiang, Linhui Qian, Xiufang Xu, Zhibo Yan

**Affiliations:** ^1^Department of General Surgery, Shandong Provincial Qianfoshan Hospital, Shandong University, Jinan, China; ^2^Department of General Surgery, The First Affiliated Hospital of Shandong First Medical University, Jinan, China; ^3^Department of Neonatal Pediatrics, Weifang Yidu Central Hospital, Qingzhou, China; ^4^Department of Anorectal Surgery, Feicheng People's Hospital, Feicheng, China; ^5^Department of Nursing, Huantai TCM Hospital, Zibo, China; ^6^Department of Hernia and Abdominal Wall Surgery, General Surgery, Qilu Hospital, Cheeloo College of Medicine, Shandong University, Jinan, China

**Keywords:** free peritoneal flap, incisional hernia, laparoscopic hernia repair, lower abdominal, marginal hernia, suprapubic hernia

## Abstract

**Background:** During lower abdominal marginal hernia repair, the peritoneal flap is routinely freed to facilitate mesh placement and closed to conclude the procedure. This procedure is generally called trans-abdominal partial extra-peritoneal (TAPE). However, the necessity of closing the free peritoneal flap is still controversial. This study aimed to investigate the safety and feasibility of leaving the free peritoneal flap *in-situ*.

**Methods:** A retrospective review was conducted on 68 patients (16 male, 52 female) who underwent laparoscopic hernia repair between June 2014 and March 2021. Patients were diagnosed as the lower abdominal hernia and all required freeing the peritoneal flap during the operation. Patients were divided into 2 groups: one group was TAPE group with the closed free peritoneal flap, another group left the free peritoneal flap unclosed. Analyses were performed to compare both intraoperative parameters and postoperative complications.

**Results:** There were no significant differences in demographic, comorbidity, hernia characteristics and ASA classification. The intra-operative bleeding volume, visceral injury, hospital stay, urinary retention, visual analog scale (VAS) score, dysuria, intestinal obstruction, surgical site infection, mesh infection, recurrence rate and hospital stay were similar among the two groups. Mean operative time of the flap closing procedure was higher than for patients with the free peritoneal flap left *in-situ* (*p* = 0.002). Comparisons of postoperative complications showed flap closure resulted in a higher incidence of seroma formation (*p* = 0.005).

**Conclusion:** Providing a barrier-coated mesh is used during laparoscopic lower abdominal marginal hernia repair, it is safe to leave the free peritoneal flap *in-situ* and this approach may prevent the occurrence of seromas.

## Introduction

**Figure 1:**
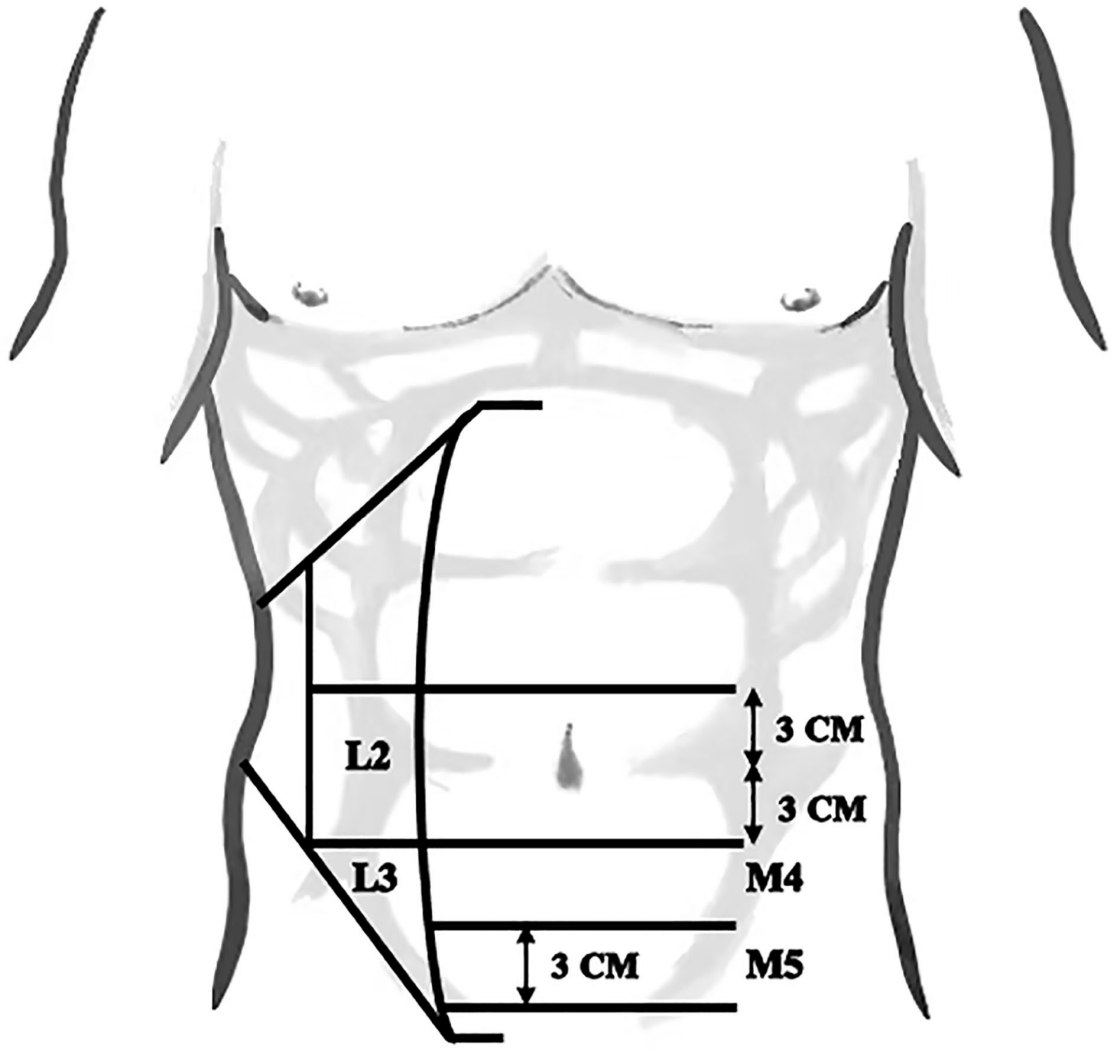
To classify the lower abdominal marginal hernia, four zones were defined according to the European Hernia Society (EHS) classification.

As a special type of hernia, there is no consensus on the definition of lower abdominal marginal hernia. Thus far, lower abdominal marginal incisional hernia and suprapubic hernia are considered subtypes of lower abdominal marginal hernia (1, 2). Based on the European Hernia Society (EHS) classification, M5, L3 or partial M4 and L2 needing a free peritoneal flap during repair belong to the lower abdominal marginal hernia classification (Figure 1) (3, 4). Among these types, suprapubic hernias, which are located <3–4 cm on the pubic arch in the midline are most commonly seen (5). Additionally, incisional hernias occurring in these areas are also regarded as lower abdominal marginal hernias (1, 6).

With respect to lower abdominal marginal hernia, the most challenging aspect for surgery involves wall defects of the abdominal borders. Difficulties can arise because the borders are the weakest points of the abdominal wall and represent the most common location of recurrence after mesh repair of incisional hernia (1). Here proximity to important anatomical structures including the urinary bladder and iliac vessels usually leads to inadequate mesh overlaps. Moreover, high abdominal wall pressure occurs in the erect position, thus, the repair technique is challenging with recurrences often following suprapubic hernia surgery (5, 7–9).

Laparoscopic incisional hernia repair first emerged in 1993 (10), with lower recurrence rates, less abdominal pain and complications compared with open repair. This technique allows surgeons better visualization of the hernial defect, and also permits improved mesh overlap (11, 12). Laparoscopy provides several key advantages to avoid recurrence, freeing the peritoneal flap, exposing the pubic comb ligament, and fixing the inferior edge of the mesh to the bony structure of the lower edge of the abdomen (13). In 2011, Sharma et al. (14) first described the trans-abdominal partial extra-peritoneal (TAPE) technique, a laparoscopic approach for the treatment of the suprapubic hernia where the hernia defect was closed and repaired with mesh partially covered by the free peritoneal flap. This technique was subsequently adopted and reported by other surgeons (13, 15). However, closing the free peritoneal flap requires skilled surgical technique, and previous research reports when using barrier-coated mesh, there is no need to re-approximate the free peritoneal flap (16). Moreover, incomplete closure of the flap may develop openings that result in internal hernias (17–19). Furthermore, the space between the barrier-coated mesh and free peritoneal flap may facilitate the formation of seroma (20, 21). Despite these known issues, no literature has previously reported the feasibility of leaving the free peritoneal flap unclosed.

Thus, the purpose of this study was to retrospectively analyze the clinical data associated with our lower abdominal marginal hernia patients and to compare the results of closed and unclosed free peritoneal flap during laparoscopic hernia repair.

## Patients and Methods

### Design

The retrospective study included 68 patients with lower abdominal marginal hernia who underwent laparoscopic hernia repair in Qilu Hospital of Shandong University from Jun 2014 to March 2021. All surgeries were performed by three experienced surgeons, each with experience of more than 400 laparoscopic hernia repairs (22). The patients were divided into either closed or unclosed groups based on the disposition of the free peritoneal flap. The inclusion and exclusion criteria are listed in Table 1. Patient outcomes were analyzed according to demographic and clinical variables.

**Table 1:** Inclusion and exclusion criteria.

**Inclusion criteria**
Adult patients with lower abdominal marginal hernia classified as M5, L3 or partial M4 and L2 which need to free peritoneal during the operation according to the European Hernia Society (EHS) classification.
Tolerant to surgery and anesthesia.
Unilateral hernia.
**Exclusive criteria**
Patients with contraindications of laparoscopic surgery, such as:
Poor cardiopulmonary function or any vital organ dysfunction.
Blood coagulation disorder.
Severe hemorrhagic disease.
Intolerant to surgery and anesthesia.
Patients underwent emergency surgery.

### Patient Demographics and Characteristics

We collected the following patient variables: age, gender, body mass index (BMI), pathogenic factors, previous surgery, smoking, ASA classification, follow-up time, comorbidities including coronary heart disease (CHD), diabetes mellitus and hypertension. Procedural parameters included hernia duration, hernia side type and the amount of recurrent hernia.

### Operative Procedure

Laparoscopic lower abdominal marginal hernia repair was performed according to TAPE technique with modifications similar to those described by Fan et al. (13, 14). After general anesthesia, the patient was placed in supine position and a pneumoperitoneum created at 12 mmHg with a Veress needle. A 10 mm trocar was placed for insertion of the 30° laparoscope and two to three 5 mm trocars were inserted for preperitoneal dissection, adhesiolysis, placement and fixation of mesh. After careful exploration of the abdominal cavity, the standardized approach was to precisely dissect the adhesions around the hernial ring and reduce or excise the contents of the hernia. The hernia ring was then interruptedly sutured by PDS-II and knotless polydioxanone. Next, the pubic bladder space was dissociated and prepared, the bilateral Cooper's ligaments were exposed to ensure mesh placement and fixation, and the mesh was placed in the abdominal with the lower end of the mesh inserted into the Retzius space. The lower edge of the mesh was fixed to the pubic comb and Cooper's ligament. The mesh types and surgical fixations used are shown in Tables 2, 3, respectively. Thereafter, the free peritoneal flap was continuously sutured and closed or unclosed based on surgeon preference. Afterwards, a drainage tube was routinely placed in the abdominal cavity to reduce the incidence of seroma. Finally, all trocars were removed and the abdomen was deflated. When it was difficult to close the hernia ring through laparoscopy, a suitable size incision was made on the top of the hernial ring. After careful exploration and lysis of adhesions, the hernial ring was closed by continuous suture under the open state. The mesh was then introduced and fixed through laparoscopy. Intra-operative bleeding volume was routinely recorded during the operation. When the amount of bleeding was small, it was measured by the suction volume and or weight of the gauze used during the operation.

**Table 2:** Mesh brands in two groups.

**Mesh brands**	**Closed free peritoneal flap (*n =* 25)**	**Unclosed free peritoneal flap (*n =* 43)**	***p* value**
Bard ® Composix E/X Mesh (*n*/%)	0/0.0	1/2.3	1
Covidien ® Parietex composite (*n*/%)	4/16.0	10/23.3	0.548
Bard ® Sepramesh (*n*/%)	21/84.0	32/74.4	0.545

*Data are expressed as numbers and percentages*.

**Table 3:** Fixation of mesh in patients who underwent laparoscopic hernia repair.

**Fixed mode**	**Patients (*n =* 68)**
Auto-suture tacks (*n*/%)	59/86.8
Medical adhesive (*n*/%)	3/4.4
Absorbable suture (*n*/%)	6/8.8

*Data are expressed as numbers and percentages*.

### Statistical Analysis

Qualitative data, percentages and frequencies were calculated, and the Fisher's exact test used to compare the two groups. Quantitative data were represented by the mean ± standard deviation (SD). The student's t test was used to compare continuous variables, whereas the Fisher's test was used for categorical variables. The “p” values for hypothesis testing were considered statistically significant when p <0.05. All calculations were performed using SPSS 26.0 (IBM SPSS Statistics, IBM Corp., USA).

## Results

A total of 68 patients with lower abdominal marginal hernia underwent laparoscopic hernia repair in Qilu Hospital of Shandong University. Patient demographics, comorbidities, hernia characteristics and treatments are summarized in Table 4. Among these cases, 16 (16.7%) were male and 52 (83.3%) were female, ranging from 32 to 86 years (mean 61.4 ± 11.5). The free peritoneal flap was closed in 25 patients and not closed in 43 patients with a mean follow-up time of 60.5 ± 25.5 months. Of the 68 patients, 31 (45.6%) patients had undergone bowel related surgery, 17 (25.0%) patients had undergone gynecological surgery, 5 (7.4%) patients had undergone urological surgery, and 10 (15.9%) patients had undergone prior incisional hernia repair. Only 4 (5.9%) patients had undergone prior laparoscopic surgery. Age, gender distribution, BMI, and ASA score were comparable between the two groups with no significant differences recorded for any parameters using an unpaired Student's *t*-test or Fisher's exact test as required (Table 5).

**Table 4:** Comparison of demographics, comorbidities and hernia characteristics of patients in two groups.

**Variables**	**Closed free peritoneal**	**Unclosed free peritoneal**	***p-*value**
	**flap (*n =* 25)**	**flap (*n =* 43)**	
Age (years)	59.3 ± 12.2	62.7 ± 11.0	0.241
**Gender (** ***n*** **/%)**			0.944
Male	6/24.0	10/23.3	
Female	19/76.0	33/76.7	
BMI (kg/m^2^)	26.6 ± 3.2	25.9 ± 3.9	0.44
**Hernia duration (** ***n*** **/%)**			0.902
≤1 months	2/8.0	2/4.7	
≤6 months	6/24.0	12/27.9	
≤1 years	8/32.0	16/37.2	
≤5 years	6/24.0	7/16.3	
>5 years	3/12.0	6/13.9	
**Hernia side (** ***n*** **/%)**			0.128
Left flank	6/24.0	3/7.0	
Right flank	10/40.0	19/44.2	
Median	9/36.0	21/48.8	
**Pathogenic factors (** ***n*** **/%)**			
Incision infection	1/4.0	1/2.3	1
Chronic constipation	0/0.0	3/7.0	0.292
Chronic cough	2/8.0	3/7.0	0.876
**Previous surgery (** ***n*** **/%)**			0.137
Open	22/88.0	42/97.7	
Laparoscopic	3/12.0	1/2.3	
**Comorbidities (** ***n*** **/%)**			
CHD	3/12.0	7/16.3	0.735
Diabetes	3/12.0	9/20.9	0.513
Hypertension	7/28.0	20/46.5	0.133
Smoking (*n*/%)	3/12.0	6/14.0	1
Recurrent hernia (*n*/%)	6/24.0	4/9.3	0.066
**ASA**			0.57
<	17/68.0	32/74.4	
≥	8/32.0	11/25.6	
Follow-up time (months)	58.8 ± 21.6	61.4 ± 27.7	0.683

*Data are expressed as mean ± SD or as numbers and percentages*.

*BMI body mass index, CHD coronary heart disease, ASA American society of anesthesiologists*.

**Table 5:** Previous surgery in patients who underwent laparoscopic hernia repair.

**Previous surgery**	**Closed free peritoneal flap (*n =* 25)**	**Unclosed free peritoneal flap (*n =* 43)**	***p*-value**
Bowel related (*n*/%)	11/44.0	20/46.5	0.841
Gynecological (*n*/%)	5/20.0	12/27.9	0.468
Urological (*n*/%)	1/4.0	4/9.3	0.645
Incisional hernia repair (*n*/%)	6/24.0	4/9.3	0.154
Others (*n*/%)	2/8.0	3/7.0	1

*Data are expressed as numbers and percentages*.

Our analysis of perioperative outcomes revealed the two operative groups were similar in terms of hernia size, defect size, mesh size, hernial ring diameter, intra-operative bleeding volume, duration of hospital stays, the frequency of prior surgeries or frequency of hybrid surgeries during the operation (Table 6). No intra-operative visceral injury and urinary retention occurred in the two groups. However, there were significant differences between operative times of the closed and the unclosed groups, recorded as 114.0 ± 16.8 and 98.8 ± 19.4 min, respectively (*p* = 0.002). Additionally, 5 (20.0%) patients in the closed group developed postoperative seromas, significantly higher than the unclosed group where no cases occurred (*p* = 0.005). According to seroma classification guidelines (23), all five cases were classified as II or III seromas, and among of these, 3 cases were spontaneously absorbed or resolved within 3 months, while 2 cases persisted and required management by puncture.

**Table 6:** Comparison of perioperative parameters in two groups.

**Perioperative parameters**	**Closed free peritoneal**	**Unclosed free peritoneal**	***p*-value**
	**flap (*n =* 25)**	**flap (*n =* 43)**	
Hernia size (cm^2^)	118.5 ± 104.6	94.4 ± 82.4	0.297
Defect size (cm^2^)	90.2 ± 79.2	88.7 ± 77.2	0.943
Mesh size (cm^2^)	301.4 ± 94.0	331.1 ± 148.4	0.372
Diameter of hernia ring (cm)	6.2 ± 3.1	6.7 ± 3.4	0.536
Operative time (min)	114.0 ± 16.8	98.8 ± 19.4	0.002^*^
Intra-operative bleeding volume (ml)	21.2 ± 10.1	28.7 ± 18.4	0.065
Intra-operative visceral injury (*n*/%)	0/0.0	0/0.0	
Hospital stay (days)	12.9 ± 4.5	13.1 ± 4.1	0.809
Seroma (*n*/%)	5/20.0	0/0.0	0.005^#^
Urinary retention (*n*/%)	0/0.0	0/0.0	
**Combined with other operations (** ***n*** **/%)**			0.981
Bowel related	1/4.0	2/4.7	
Hernia repair	1/4.0	1/2.3	
Others	1/4.0	2/4.7	
Hybrid surgery (*n*/%)	6/24.0	8/18.6	0.596

*Data are expressed as mean ± SD or as numbers and percentages*.

# *Fisher's exact test was used and a p-value of < 0.05 is considered significant*.

* *Student's t test was used and a p-value of < 0.05 is considered significant*.

The VAS score of all patients, duration of postoperative pain (days), recurrence and other complications were recorded after a mean follow-up period of 60.5 ± 25.5 months (Table 7). The overall occurrence of SSI, mesh infection and recurrence were 1.5% (*n* = 1), 1.5% (*n* = 1) and 3.0% (*n* = 2). Notably, other common complications including dysuria, intestinal obstruction and hematoma did not occur in either group. There were no significant differences found between the groups among these post-operative complications using Fisher's exact test.

**Table 7:** Comparison of postoperative VAS score, pain days, recurrence and other complications.

**Variables**	**Closed free peritoneal**	**Unclosed free peritoneal**	***p*-value**
	**flap (*n =* 25)**	**flap (*n =* 43)**	
**VAS (** ***n*** **/%)**			0.63
0	14/56.0	29/67.5	
≤3	10/40.0	13/30.2	
≤6	1/4.0	1/2.3	
≤10	0/0.0	0/0.0	
**Postoperative pain**			0.916
≤7 days	5/13.6	8/13.2	
≤1 months	14/59.1	22/52.6	
≤3 months	4/18.2	10/26.3	
>3 months	2/9.1	3/7.9	
Dysuria (*n*/%)	0/0.0	0/0.0	
Intestinal obstruction (*n*/%)	0/0.0	0/0.0	
SSI (*n*/%)	0/0.0	1/2.3	1
Mesh infection (*n*/%)	0/0.0	1/2.3	1
Recurrence (*n*/%)	0/0.0	2/4.7	0.528

*Data are expressed as numbers and percentages*.

*SSI, Surgical site infection*.

## Discussion

As important subtypes of lower abdominal marginal hernias, both incisional hernias and suprapubic hernias usually occur following abdominal surgery (5, 24). And suprapubic hernias are always incisional hernias (14). Successful operative repair of these hernias markedly improves patient's quality of life (25). Nonetheless, treating this type of hernia poses difficulties for surgeons since the site is very close to the bladder and there is no rectus sheath below the arcuate line leading to reduce fascial support (13). Insufficient Retzius space and fixation of mesh to impermanent structures can also result in recurrence (5). Based on these factors, the optimization of approaches to hernial procedures have become a hot topic in the surgical field. Previous reports have indicated that the TAPE technique significantly reduces the recurrence rate and mesh-induced complications of suprapubic incisional hernia (13–15). However, closing the peritoneal flap during TAPE prolongs the operative time. Notably for the trans-abdominal preperitoneal (TAPP) technique, the routine treatment for inguinal hernia, it has been shown that not closing the peritoneal flap produces shorter operative time and provides similar recurrence rates (after the preperitoneal space is dissociated and a barrier-coated mesh is placed) (26, 27).

On this basis, we investigated the safety and feasibility of leaving the free peritoneal flap *in-situ* during laparoscopic hernia repair for lower abdominal marginal hernia. We integrated an assessment of the patient's preoperative condition, perioperative performance, short-term postoperative complications together with follow-up investigation of VAS scores, the rate of recurrence, and other long-term complications. Our study found that not closing the free peritoneal flap can shorten the operative time and reduce the incidence of seroma.

Seroma is one of the most common complications after lower abdominal marginal hernia operations. Its occurrence is related to the size of the surgical site, the size of the dead space and the placement of the drainage tube (28). The occurrence of seroma in our study was similar to previous reports (29, 30). However, we observed no postoperative seromas were formed in the unclosed group, clearly illustrating that not closing the free peritoneal flap may be advantageous in preventing seroma. In the closed group, there were 5 (20.0%) patients with postoperative seromas. All 5 seromas occurred between the free peritoneal flap and the mesh, likely because of detachment of anti-adhesion layer of the coated-barrier mesh, or the exudate of the peritoneal flap was not fully absorbed and failed to drain out. Two cases were eventually resolved by puncture treatment without inducing mesh infection. It is worth noting that although closing the peritoneal flap and leaving a space may facilitate the outflow of fluid in front of the peritoneal flap, the intestine may herniate into the front of the peritoneal flap through this space so that it may cause an intestinal obstruction.

As a risk factor of postoperative morbidities and readmission (31, 32), operative time also represents an important parameter examined in our study. Simplifying the step of closing the free peritoneal flap was expected to reduce the operative time and therefore, leaving the free peritoneal flap in place provides this advantage (26). Furthermore, Ross et al. reported that closing the free peritoneal flap with tacks produces shorter operative times (33). However, sutures are needed where the tacks fail to fix the peritoneal flap, and this frequently occurs where the peritoneum is relatively thin (34). Importantly, multiple studies have shown that the use of tacks for mesh fixation is associated with postoperative chronic pain and recurrence, particularly where a higher number of tacks are used (33, 35, 36). Thus, reducing the application of tacks may reduce the incidence of postoperative chronic pain.

Hospitalization costs also represent a very important observation parameter. Alkhoury et al. reported that the use of polypropylene mesh in laparoscopic hernia repair is cost effective (37), but this method did not significantly reduce overall operative costs. This arises because the surgical method used requires a polypropylene mesh (covered by a peritoneal flap) and a barrier-coated mesh (contacting the abdominal cavity directly). In this study, we did not find differences in cost because barrier-coated meshes were used in all operations.

Furthermore, postoperative urinary retention is another important factor to consider. The proximal relationship between hernia and the bladder means it is common to induce urinary retention and dysuria because the bladder usually needs to be freed to repair lower abdominal marginal hernias (38). It is also uncertain whether not fixing the freed bladder to the abdominal wall causes urinary retention. We commonly mobilize and carefully protect the bladder during the operation, which we consider a crucial step in the success of the operation (16). Although we do not restore bladders to their original anatomical positions, no urinary retention and dysuria were found in all of our 38 patients during follow-up.

Recurrence is always the principal problem in lower abdominal marginal hernia. The main reasons for the high rate of recurrence involve the anatomical location and body position with three critical considerations (1, 9, 13, 15, 39): (1) the anatomical location including the iliac vessels and bladder; (2) erect position; and (3) the fact that this area represents the weakest point of the abdominal wall (1, 9, 13). In our study, the recurrence rate was 2.9% (2/68 cases) with no statistical significance between the two groups seen through follow-up investigations. Our analysis therefore indicates the recurrence of lower abdominal marginal hernia was not influenced by whether or not the free peritoneal flap was closed. This conclusion is similar to reports for inguinal hernia repair where similarly, no relationship between the closure of the peritoneal flap and the rate of recurrence was observed (26). On this basis, the choice to close the free peritoneal flap should not assumption that this will affect recurrence rates. Finally, we observed only 1 (1.5%) case of incision infection and 1 case (1.5%) of mesh infection. These infections required us to change dressings and anti-infection treatments. Thus, we recommended choosing mesh with high biological activity to prevent mesh infection.

Finally, we must acknowledge the limitations of our study. Foremost, this was a single-institution retrospective review study. As a result, only 68 cases were included, which can be considered relatively low compared with other clinical studies. Furthermore, the study relied on retrospective data which could be susceptible to bias. Moreover, the follow up data was collected by telephone interview. Therefore, a more comprehensive comparison between the closure and non-closure of the free peritoneal flap will require increasing both sample size and more extensive patient follow up. Ideally, a multiple-institution randomized controlled trial will provide a definitive assessment of the advantages and disadvantages of the two techniques.

## Conclusion

In conclusion, the results of our study indicate that for laparoscopic hernia repair of lower abdominal marginal hernia, compared with the closure of the free peritoneal flap, the non-closure of the free peritoneal flap has no obvious disadvantages. This procedure reduces operative times and the occurrence of postoperative seroma. Therefore, the choice to not close the free peritoneal flap during lower abdominal marginal hernia repair procedures is safe and feasible.

## Data Availability Statement

The raw data supporting the conclusions of this article will be made available by the authors, without undue reservation.

## Author Contributions

ZY contributed to the original idea and conceptual design. QX and GZ contributed to the drafting of the work. FX and LQ provided critical review of the article. LL and XX provided final approval of the version published. All authors contributed to the article and approved the submitted version.

## Conflict of Interest

The authors declare that the research was conducted in the absence of any commercial or financial relationships that could be construed as a potential conflict of interest.

## Publisher's Note

All claims expressed in this article are solely those of the authors and do not necessarily represent those of their affiliated organizations, or those of the publisher, the editors and the reviewers. Any product that may be evaluated in this article, or claim that may be made by its manufacturer, is not guaranteed or endorsed by the publisher.
